# Development of High-Performance Enamel Coating on Grey Iron by Low-Temperature Sintering

**DOI:** 10.3390/ma11112183

**Published:** 2018-11-04

**Authors:** Dan Song, Ren Tang, Falin Yang, Yanxin Qiao, Jiapeng Sun, Jinghua Jiang, Aibin Ma

**Affiliations:** 1 College of Mechanics and Materials, Hohai University, Nanjing 210098, China; songdancharls@hhu.edu.cn (D.S.); Ren_tang@126.com (R.T.); yangfa1in@163.com (F.Y.); sun.jiap@gmail.com (J.S.); 2 Suqian Research Institute of Hohai University, Suqian 223800, China; 3 School of Materials Science and Engineering, Jiansgu University of Science and Technology, Zhenjiang 215004, China; yxqiao@just.edu.cn

**Keywords:** enamel coating, low-temperature sintering, microstructure characteristic, friction and wear property

## Abstract

In this study, we report on a low-temperature sintered enamel coating with a high-strength bonding and wear-resistance that protected a grey cast iron substrate. The SiO_2_–Al_2_O_3_–B_2_O_3_ composited prescription for the enamel coating was modified by the partial substitutions of SiO_2_ for B_2_O_3_ and alkali metals for Li_2_O. The optimized enamel coating was prepared by sintering at a relatively low temperature (730 °C) for seven minutes. Due to the composition of both the amorphous and crystalline phases, the enamel coating presented sufficient hardness and excellent wear resistance. The wear volume loss and the specific wear rate of the enamel coating were obviously lower than that of the metal substrate. The enamel coating can effectively improve the service life of the grey cast iron substrate in a complex frictional environment.

## 1. Introduction

Cast-iron overflow components, which are one of the most important pieces of hydraulic equipment, have been widely applied in the industrial field. However, the overflow component is always used in a complex environment, particularly in sand-containing waters. The overflow component suffers both corrosion and strong erosion damage, as well as interaction damage. These factors promote the destruction of the overflow component, leading to tremendous loss [1,2,3,4,5]. 

Enamel is an inorganic glass material, which, when applied in the form of a complete and dense coating, has the advantages of high hardness, corrosion resistance, and wear resistance. By covering the enamel coating, both the strength and toughness of the metallic substrate as well as the advantages of the chemical stability and mechanical properties of the enamel coating can be simultaneously achieved by the cast iron overflow component. Therefore, an enamel coating will greatly improve the surface performance and extend the service life of a cast-iron overflow component.

In the last few decades, the application of enamel-coating technology in cast-iron overflow components has been reported, including many hydraulic pieces of equipment, such as pumps, pipes, and turbine blades [6,7,8,9,10,11,12,13]. However, problems and deficiencies still exist. Due to the large amount of graphite in the cast-iron substrate, elemental carbon is prone to oxidization and vaporization during the process of high-temperature sintering, leading to the deterioration of compactness and adhesion of the enamel coating, as well as a limited coating performance. Therefore, by optimizing the enamel composition and the sintering process, the low-temperature sintering of the enamel coating can not only reduce the sintering energy consumption, but also suppress the excessive gasification of the elemental carbon in the cast-iron matrix during the sintering process as well as improve the adhesion and serviceability of the enamel coating [14,15,16,17].

In this paper, grey cast iron (Chinese standard HT200, which has the chemical composition of 3.2 wt% C, 0.6 wt% Mn, 1.6 wt% Si, 0.1 wt% S, 0.11 wt% P, and the balance of Fe) was selected as the overflow component matrix. A low-temperature sintered enamel with high-strength bonding and wear resistance was designed and prepared. The microstructure and mechanical properties of the enamel coating were studied. The friction and wear properties as well as its wear protection effect on the metal substrate were evaluated. Meanwhile, the friction and wear mechanism of the enamel coating were revealed.

## 2. Experimental Procedure

### 2.1. Coating Formation Processing

**Figure 1:**
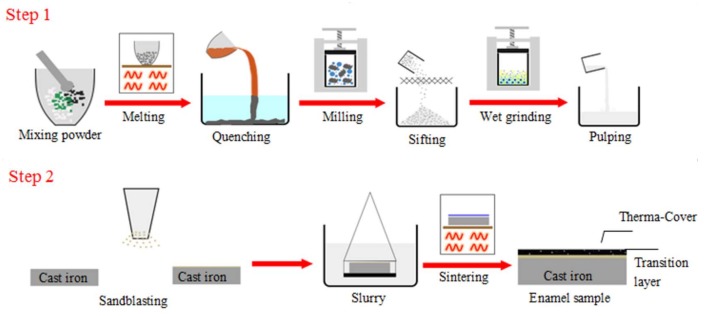
Processing of sintering enamel coating on the grey cast iron.

As illustrated in Figure 1, the enamel coating formation process includes two major steps. The first step is the enamel powder preparation, and the second step is sintering the enamel coating. The first step has three segmented processes, including enamel frit preparation, ball milling, and pulping. The chemical composition of the raw materials used for the enamel frit are listed in Table 1. In this designed SiO_2_–Al_2_O_3_–B_2_O_3_ composited prescription, some quantity of SiO_2_ was used instead of B_2_O_3_, and some of the alkali metals were used instead of Li_2_O. However, the excessive use of B_2_O_3_ and Li_2_O will cause a much larger thermal expansion coefficient of the enamel coating than that of the metal substrate, which results in large areas of flaking and voiding in the coating during the cooling phase. Through proper modification, a complete and compact enamel coating can be sintered at a relatively lower temperature.

The raw materials were uniformly mixed and melted in an electronic furnace. They were first heated at a temperature of 700 °C for 50 min to achieve the complete decomposition of Na_2_CO_3_ and Li_2_CO_3_. Then, the raw materials were further heated at 1150~1200 °C for 60 min until they were completely melted. The melt was quenched quickly in water to obtain the enamel frit and were then dried completely in hot air. The obtained enamel frit was further milled with a corundum grinding ball in a planetary ball milling machine for 12 h at a grinding speed of 300 r/min, then sieved through a 150-mesh sieve to achieve a uniform and fine enamel powder. The obtained enamel slurry was mixed with mill additives according to the chemical composition listed in the Table 2, then wet grinded with a corundum grinding ball for 1 h, and was further precipitated into a suspended enamel slurry.

The second step also has three segmented processes, including substrate surface pretreatment, hanging pulp, and sintering. The grey cast iron sample with a size of 20 × 20 × 2 mm was mechanically polished and sandblasted to create a fresh surface and increase the surface roughness, which enlarges the contact area between the substrate and the enamel slurry. The pretreated metal substrate was completely immersed in the enamel slurry, shaken evenly for 30 s, then carefully removed. It was ensured that the enamel slurry was evenly distributed on the substrate with a moderate thickness. No wrinkles or accumulation on the surface could be found. Then, the pulped samples were dried at 100 °C for 1 h. The sample was finally sintered at a set heating temperature and time. To optimize the sintering parameter, the heat temperatures were set from 700 °C to 760 °C, with intervals of 10 °C and the heating time was set at 3, 5, 7, 9, 11, and 13 min. The sintered samples were cooled in the furnace to 350 °C and were further cooled in the air to room temperature.

### 2.2. Microstructure Observation

The optical microstructure (OM) of the grey cast iron was observed by the optical microscope (Olympus BX51M, Tokyo, Japan). Samples for OM observation were mechanically polished and etched by the 4% nital solution. The microstructure characteristic of the enamel coating was observed via a scanning electron microscope (SEM, Hitachi S4800, Tokyo, Japan). The cross-sectional chemical composition analysis of the enamel coating and substrate was characterized by an energy dispersive x-ray spectrometer (EDS, OXFORD instrument, Oxford, Oxfordshire, UK). The phase composite of the enamel coating was performed while using a Bruker D8 Advance diffractometer (Bruker AXS, Karlsruhe, Germany) with Cu Kα radiation. The θ-2θ diffraction patterns were scanned from 10° to 90° with a scanning rate of 2°∙min^−1^.

### 2.3. Mechanical Property Testing

The micro-hardness of the enamel coatings was tested via an HXD-1000TC micro-hardness tester (Shanghai Optical Instrument Factory, Shanghai, China). The load was 200 g, with a load time of 15 s. Five parallel samples for both enamel coating and grey cast iron was tested. Five different locations were tested to obtain the average micro-hardness value of each parallel sample. The bonding stress between the enamel coating and the metal substrate was evaluated according to the ASTM D4541–09 standard [18]. As the tensile sample for bonding stress testing, the enamel coating sample was stacked to the chuck via glue. It should be emphasized that the glue strength was much higher than the bonding stress between the enamel coating and the metal substrate. The area where the coating was bonded to the metal substrate was 20 × 20 mm^2^ and was tested at room temperature with a tensile speed of 0.18 mm/min. The bonding stress was calculated via the following equation [19]:

(1) $$R_{b}=F_{break}/S$$

where *R_b_* is the bonding stress between the enamel coating and the metal substrate (unit: N/mm^2^); *F_break_* is the tested load value when the enamel coating was pulled off (unit: N); and, *S* is the area where the coating is bonded to the metal substrate (unit: mm^2^).

### 2.4. Wear-Resisting Property Testing

The wear tests were conducted at the ball-on-disc mode via an MFT-3000 tester (Rtec, San Jose, CA, USA), of which the sample was fixed on the disc and a tungsten carbide (WC) ball with diameter of 12.7 mm was used. During the testing, the WC ball kept static and the reciprocating linear movement of the disc drove the friction between the WC ball and the tested sample. The friction and wear tests were performed in the dry condition without humidity control at room temperature. Two controlling parameters, the applied load and the sliding speed, was tested to evaluate the wear resistance of the enamel coating and the grey cast iron. The detailed testing parameters are listed in Table 3. The tested data were provided by the attached software (Mech Stress Stage-Short), and the friction coefficient curves were drawn based on the data. After the tests, the samples were cleaned with acetone, and dried completely. The wear trace micro-morphologies were observed via SEM, and the wear volume loss was calculated. The specific wear rate is an important indicator for measuring the wear resistance of the materials, which can be calculated according to the following equation [19]:

(2) K=V/(sF)

where *K* is the wear rate (mm^3^∙N^−1^∙M^−1^), *V* is the wear volume (mm^3^), *s* is the sliding distance (m), and *F* is load value (N). Meanwhile, the wear volume *V* can be artificially divided into two parts. The first part (*V*_1_) can be calculated by the multiplying the sliding stroke (*L*, the set value is 1 mm) and the cross-sectional area of the WC ball embedded in the wear tested sample, which can be theoretically calculated according to the equation (3). The second part (*V*_2_) can be regarded as the volume of the WC ball embedded in the wear tested sample, which can be theoretically calculated according to the equation (3). Following are the detailed equations, where r is the WC ball radius (mm) and d is the width of the wear trace left after the friction test (mm).

(3) $$V_{1}=\pi {\left(r-\sqrt{r^{2}-0.25d^{2}}\right)}^{2}\left(r-\frac{\left(r-\sqrt{r^{2}-0.25d^{2}}\right)}{3}\right)$$

(4) $$V_{2}=L\left(\arcsin\left(\frac{d}{2r}\right)r^{2}-0.5d\sqrt{r^{2}-0.25d^{2}}\right)$$

(5) $$V=V_{1}+V_{2}$$

## 3. Results and Discussion

### 3.1. Macro-Morphologies and Microstructure Characteristic of the Enamel Coating

**Figure 2:**
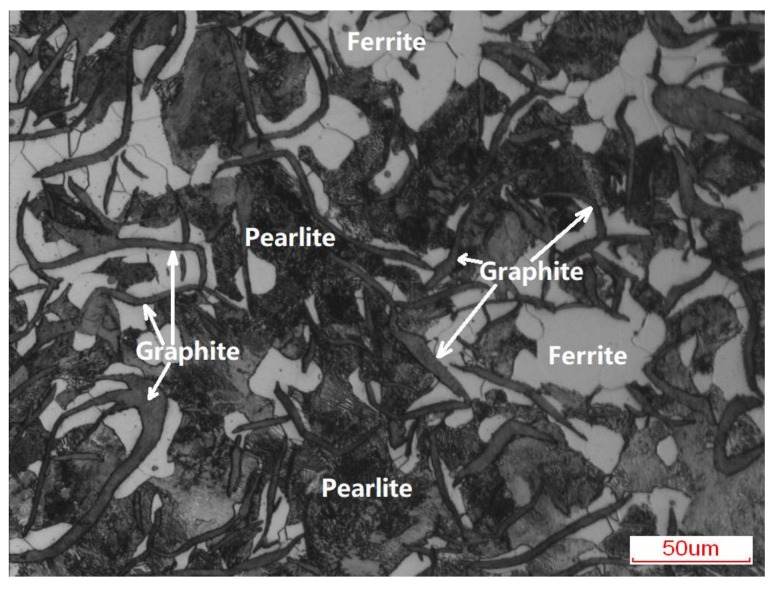
Optical microstructure of the grey cast iron substrate.

Figure 2 is the typical optical microstructure of the grey cast iron substrate. As marked by the white arrows, the microstructure of the grey cast iron was composed of the pearlite, ferrite, and graphite. The pearlite and ferrite constituted the matrix, of which 80% was the pearlite. Based on the statistical analysis of the OM images obtained from three parallel samples, the proportion of the graphite was about 15%. Most of the graphite had the lamellar morphology, and very few possessed the dendrite point-like morphology.

**Figure 3:**
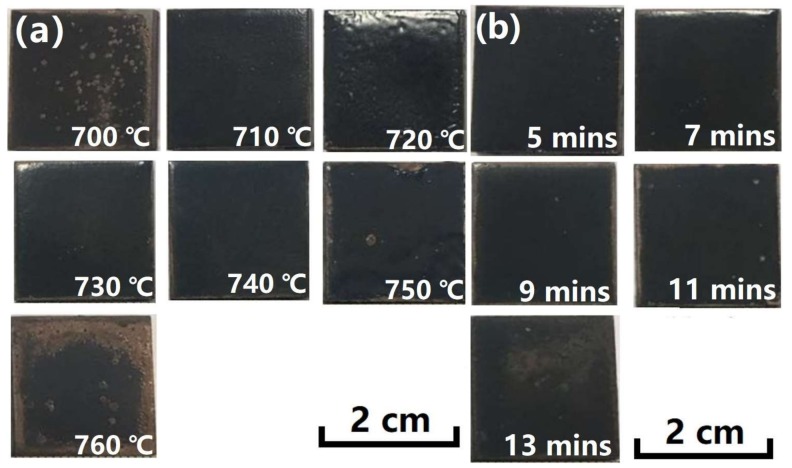
Optical macro-morphologies of the enamel coating fabricated at different sintering temperature and time. (**a**) Samples sintered at different temperature for 5 min; and, (**b**) samples sintered at 730 °C for different time.

The enamel coating was prepared by sintering at a relatively high temperature. The excellent performance of the coating is sensitive to the sintering temperature and holding time; therefore, it is necessary to determine the optimum sintering process through a large number of tests. Figure 3a shows the optical macro-morphologies of the enamel coating fabricated at different sintering temperatures for five minutes, and Figure 3b shows the samples that were sintered at 730 °C for different times. In this study, the coating performance as well as the optimum sintering process was initially judged by the macro coating morphologies. Substantial differences could easily be found in the macro morphologies of the coating that was sintered at different heating temperatures; coatings that presented a typical loose porous morphology showed an under-burned state due to the low sintering temperatures of 700 °C, 710 °C, and 720 °C. As the samples were sintered at much higher temperatures (750 °C and 760 °C), defects, such as wrinkles and charred spots, occurred on the coating surface, presenting a typical over-burned state. It is note-worthy that coatings sintered at the 730 °C and 740 °C had complete and perfect macro-morphologies. In light of the low energy consumption during sintering, the lower sintering temperature (730 °C) was selected in the following investigation to reveal the effect of heating time on the enamel coating sintering. Significant differences could also be found in the macro morphologies of the coatings sintered for different heating times. The short-time (five minutes) sintered enamel coating had obvious pinhole-like macro holes and poor gloss. When the sintering time was prolonged to 11 and 13 min, the enamel coating suffered obvious shrinkage. The seven minute sintered enamel coating appeared to be the best.

**Figure 4:**
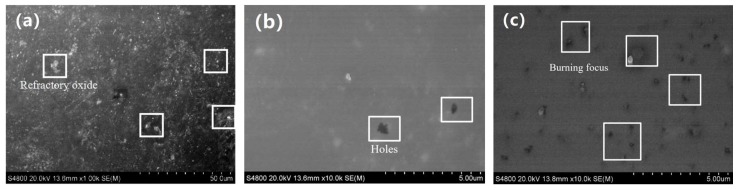
Scanning electron microscope (SEM) micro-morphologies of the enamel coating sintered at different temperature for 5 min. (**a**) is 700 °C, (**b**) is 730 °C, and (**c**) is 760 °C.

SEM micro-morphologies of the enamel coating were used to further determine the sintering results. Figure 4a shows the micro-morphology of the enamel coating sintered at 700 °C for five minutes. Clearly, due to the low sintering temperature, the glass frit and the mill additives were not completely melted, leading to the under-burning state of the coating. In this situation, the enamel had high viscosity, large surface tension, and poor fluidity. Meanwhile, since the sintering temperature was too low, the sintering force was insufficient, and the gas bubbles that were generated by the reaction in the enamel coating had difficulty in completely escaping during the cooling process. As a result, defects such as holes may appear on the enamel coating surface, which directly affects the protection effect of the coating on the substrate. Figure 4b shows the micro-morphology of the enamel coating sintered at 730 °C for five minutes. When compared to Figure 4b, the un-melted glass frit and the mill additives were dramatically decreased in the 730 °C-sintered coating, where only quite limited micro-holes could be found. Due to an elevated sintering temperature, the viscosity of the enamel was reduced, and the bubbles generated during the sintering process could escape smoothly. Meanwhile, the pinholes left by the gas escape will be quickly filled by the fully molten enamel, thereby forming a complete enamel coating. As the sintering temperature was further elevated to 760 °C, the enamel coating presented a typical over-burning phenomenon. As seen in the SEM micro-morphologies in Figure 4c, the holes and black ablation points increased significantly. Due to the higher temperature, excess iron oxide was melted in the enamel, which reduced the melting point of the enamel, leading to an over-burning of the enamel coating [20].

**Figure 5:**
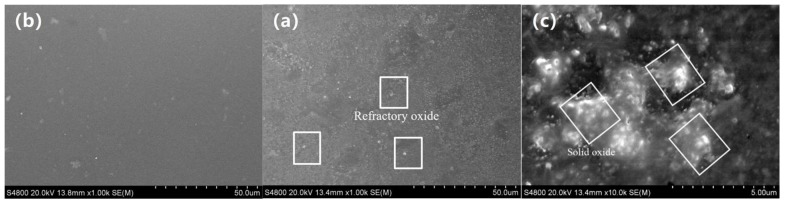
SEM micro-morphologies of the enamel coating sintered at 730 °C for different time. (**a**) is 5 min, (**b**) is 7 min, and (**c**) is 11 min.

Figure 5a–c shows the SEM micro-morphologies of the enamel coating sintered at 730 °C for five minutes, seven minutes, and 11 min, respectively. When sintered for five minutes, a certain number of un-melted refractory oxides resided in the coating, presenting an under-burning state. When the sintering time was prolonged to seven minutes, a more sufficient chemical reaction of the components of the enamel raw material could be achieved. Meanwhile, the longer the contact time between the molten enamel and the metal substrate, a more uniformly spread enamel coating could be obtained. In this situation, no micro-defects could be found, presenting a nearly perfect microstructure characteristic of the enamel coating. As the sintering time further prolonged to 11 min, the typical over-burning phenomenon could be seen in the micro-morphologies shown in Figure 5c. Due to the extensive sintering time, excess solid glaze powders were melted into a liquid state, causing a part of the lighter un-melted solid oxide to float on the surface of the coating. Meanwhile, a large amount of thermal stress accumulates in the coating during the long-duration sintering process, leading to the coating collapsing during the cooling processing.

Based on the macro and micro coating morphologies observations, as well as the consideration of the lower energy consumption, one could conclude that the optimized sintering process of the enamel coating was 730 °C for seven minutes.

**Figure 6:**
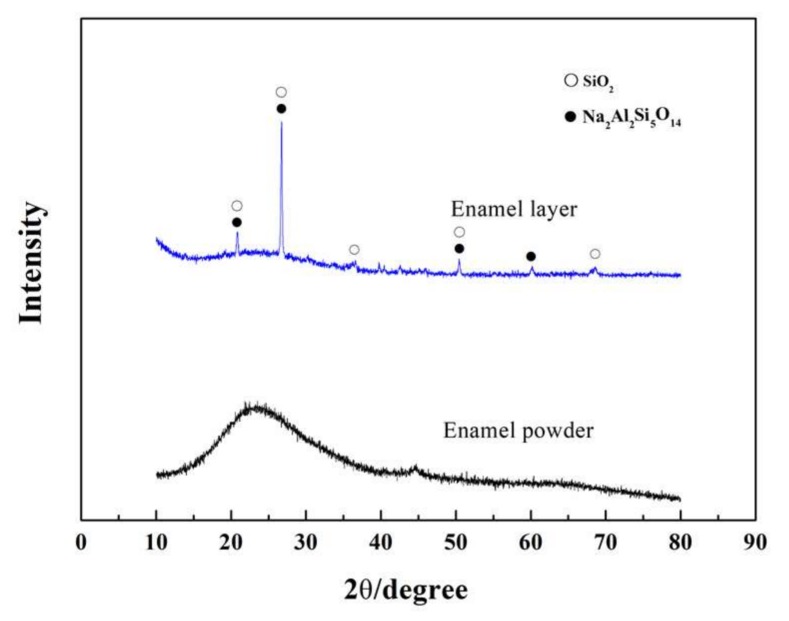
XRD patterns of the enamel powder and enamel coating.

XRD was conducted to analyze the phase composition of the optimized enamel coating, which was sintered at 730 °C for seven minutes. As seen in Figure 6, the peak position of the XRD spectrum of the enamel glaze powder was chaotic, and there was no obvious characteristic peak, indicating that the enamel glaze powder was a typical glass phase. In contrast, the XRD spectrum of the enamel coating was a diffuse peak between 25° and 35°, and a sharp crystalline peak was superimposed on the diffuse peak. Judging from the characteristic of this spectrum, one could infer that the phase composition of the studied enamel coating was composed by both amorphous and crystalline structures. After a comparison with the standard card, the crystal substance was determined to be Na_2_Al_2_Si_5_O_14_ (Na_2_SiO_3_·Al_2_SiO_5_·3SiO_2_). According to reference [21], the raw materials SiO_2_, Al_2_O_3_, and Na_2_O undergo a chemical reaction during the sintering process and finally precipitate into Na_2_Al_2_Si_5_O_14_ crystals during the cooling phase. 

**Figure 7:**
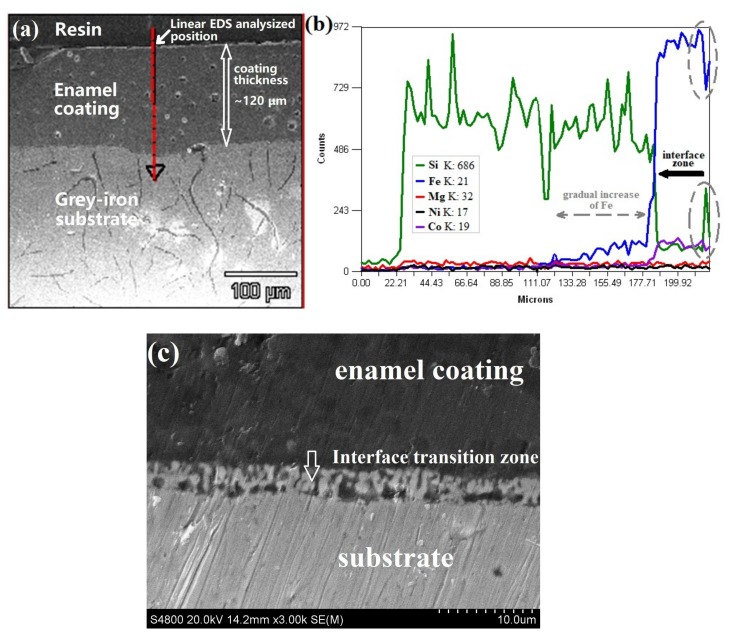
SEM morphologies (**a**) and linear energy dispersive x-ray spectrometer (EDS) analysis (**b**) of the cross-sectional view of the enamel coating, and the coating/substrate interface morphologies (**c**).

Figure 7a shows the cross-sectional SEM morphologies of the enamel coating and the substrate obtained from the optimized sintering parameter. The coating had thickness of 120~150 μm, which was uniform, dense, and complete, presenting no micro cracks in both the internal coating and the coating/substrate interface. However, limited micro holes could be found in the coating. The EDS linear analysis of the cross-sectional view of the coated sample is shown in Figure 7b, and the analysized position is marked by the white arrow in Figure 7a. The linear EDS analysis was carried out from the resin, through the enamel coating and into the substrate. As marked by the black long arrow in the curves, there were sharp alternation in the chemical composition, especially the Si and Fe content. One can believe that this sharp change was typically evident for the coating/substrate interface. Meanwhile, judged from the gradually increased of the Fe content in the coating nearing to the interface (marked as the grey dash line with two-way arrows), the obvious diffusion of the Fe element from the iron substrate into the enamel coating was validated. On the other hand, the Si, Ni, and Co elements in the enamel coating diffused significantly into the deeper areas of the metal substrate (as marked by the dashed ellipse of the element distribution curves). Detailed interface morphologies were observed by SEM with higher magnification, that an obvious zigzag-shaped interface transition zone with approximately thickness of 11 µm were clearly observed, indicating the mutual penetration phenomenon of both the coating and substrate. Based on the SEM observation and EDS analysis, one can see that a metallurgical bond has been achieved between the cast iron substrate and the enamel coating of the optimized sample.

### 3.2. Mechanical Properties of the Enamel Coating

**Figure 8:**
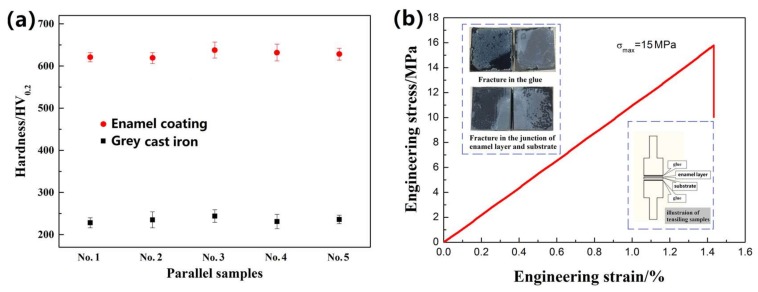
Mechanical property of the samples. (**a**) The micro-hardness of the enamel coating and grey cast iron substrate. (**b**) The bonding stress between the enamel coating and the substrate.

Figure 8a shows the micro-hardness values of the enamel coating and the grey cast iron substrate, which are respectively obtained from 5 parallel samples. The micro-hardness of the enamel coating was approximately 630 HV_0.2_, which was approximately 2.7 times of the value of the metal substrate. Its hardness is a reflection of the firmness of its internal structure, and it mainly depends on the type and strength of its internal chemical bonds. Among them, the covalent bond type has the highest hardness, then the ionic bond, the metal bond, and finally, the molecular bond. The valence state of the atom and the spacing of the atoms determine the strength of the chemical bond and are therefore an important factor in determining the hardness of the material. The chemical bonds of the enamel coating mainly have ionic bonds and covalent bonds [22]. Therefore, the enamel coating has a higher hardness than the metal substrate.

Figure 8b shows the representative engineering tensile stress–strain curves of the coated sample, which were used to evaluate the bonding stress of the enamel coating and substrate. The illustration of the samples for tensile test and the fractured sample morphologies were also provided. Through the curves, we find that the fracture strength is approximately 15 MPa, which represents the tested bonding stress of the enamel coating. As illustrated in the upper-left corner of Figure 8b, two fracture morphologies occurred after the tensile test. One occurred at the glue, and the other occurred at the junction of the coating and the metal substrate. Herein, the stress–strain curves, as well as the bonding stress value, were obtained from the coated sample fractured at the junction of the coating and the metal substrate.

### 3.3. Wear-Resisting Property of the Enamel Coating

**Figure 9:**
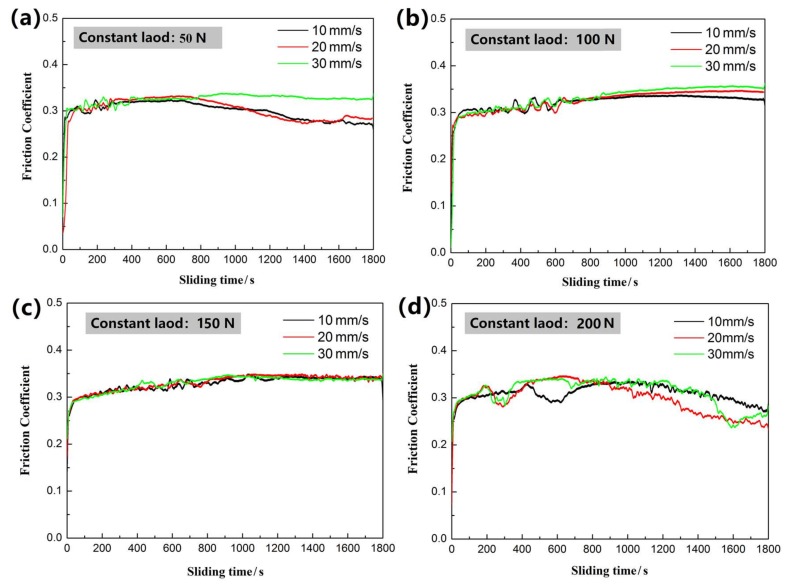
The friction coefficient curves tested at the different load and sliding speed. (**a**–**d**) are 50 N, 100 N, 150 N, and 200 N, respectively.

Figure 9 presents the friction coefficient curves tested at different constant loads (50 N~200 N) and different sliding speeds (10, 20, and 30 mm/s). Generally, the friction coefficient curve can be roughly divided into three stages: the running-in period, the transition period, and the stabilization period [15]. In the running-in period, the friction coefficient increases rapidly with time. Initially, the protective film on the surface of the friction pair acts as a lubricant when it comes into contact with the substrate, so the friction coefficient is low. However, as the sliding speed increases, the substrate directly contacts the friction pair, causing a rapid increase in the friction coefficient. During this period, the friction coefficient fluctuates greatly, which may be related to factors, such as wear debris, hard material peeling, and material adhesion during the friction process. After a period of fluctuation, the friction coefficient tends to a certain value. This outcome may be because the surface temperature of the material increases continuously during the dry friction process, so oxidative wear occurs on the surface of the material, and the friction coefficient is stabilized due to the balance of the generation and overflow of wear debris.

**Figure 10:**
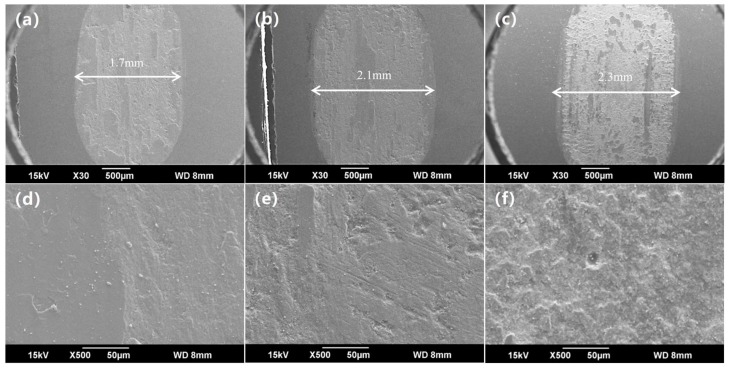
SEM image of the wear trace under the constant load 50 N with different sliding speed. (**a**–**c**) are the low-magnification images tested at 10 mm/s, 20 mm/s, and 30 mm/s; and, (**d**–**f**) are the corresponding high-magnification images.

As seen in Figure 9a, at a load of 50 N and a sliding speed of 30 mm/s, the enamel coating reached a stable period approximately 420 s into the friction test with a friction coefficient of 0.32. When the coating was tested at the lower speeds of 10 mm/s and 20 mm/s, the friction coefficient was stable at 0.27. The obvious difference in the friction coefficient of the enamel coating was caused by its surface unevenness. Only the local bulge was in contact with the tungsten carbide (WC) ball. As the sliding speed increased, the contact area increased, leading to an increased friction coefficient. As seen in the SEM image of the wear trace under a constant load of 50 N with different testing velocities, as presented in Figure 10, the wear trace became rougher and wider as the sliding speed increased. For the sample that was tested at a speed of 20 mm/s, its wear trace width increased 1.24 times when compared to that of the sample that was tested at a speed of 10 mm/s. Meanwhile, the roughness of the surface was obviously increased, accompanied by minor brittle exfoliation. As the speed increased to 30 mm/s, the wear trace width was further enlarged to 2.3 mm, and there was obvious brittle flaking around the wear trace. This phenomenon is mainly caused by the essence of brittle materials in the enamel coating. In the friction and wear test, due to plastic deformation and the bubble structure inside the coating, micro-cracks were generated in some stress-concentrated micro-domains. When the micro-cracks expand to a certain size and the external stress is larger than the enamel coating, brittle exfoliation finally occurs in the enamel coating. Moreover, as the friction test continues, the wear debris exposed on the interface, due to its irregular shape and high hardness, will be subjected to a stress transfer to the surrounding enamel coating, causing plastic deformation thereof and resulting in micro-cracks at the interface. Under the continuous load effect, as the deformation increases, severe plastic deformation occurs on the surface of the enamel coating and it forms a flaky shedding, which appears as a braided structure in the figure [23,24].

**Figure 11:**
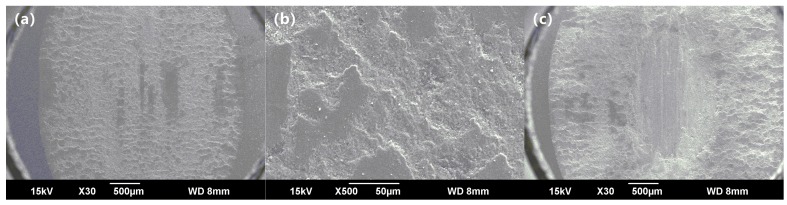
SEM image of the wear trace under the different load and sliding speed. (**a**) and (**b**) are the images tested at 100 N and 30 mm/s, respectively; and, (**c**) is the image tested at 200 N and 10 mm/s.

Figure 9b,c present the friction coefficient curves tested at the constant loads of 100 N and 150 N, respectively. After the running-in period and the transition period, the friction coefficient of the enamel coating was stabilized at 0.32~0.34. Figure 11a,b show the SEM friction and wear morphologies of the coating sample tested in the 100 N load at the speed of 30 mm/s, respectively. Clearly, due to the increased load, the wear trace width of this sample was increased by 1.57 times when compared with the sample that was tested at the same speed but with a smaller load (50 N). Meanwhile, the wear traces seemed to be much rougher, presenting fragile flaking exfoliation. The above phenomenon indicates that the micro-cracks of the enamel coating increased with an increase of the load, leading to a large piece of exfoliation [25,26]. As the load further increased to 200 N, the friction coefficient of the enamel coating was always in an unstable state (see the curves in Figure 9d). Judging from the SEM morphologies of the sample tested at a load of 200 N and speed of 10 mm/s (see Figure 11c), there were several furrows that were caused by the friction between the tungsten carbide (WC) and metal substrate, providing direct evidence that the enamel coating had been worn through under the repeated stresses of the large load. Due to the large amount of wear debris, the friction coefficient of this sample was always in an unstable state [27]. 

**Figure 12:**
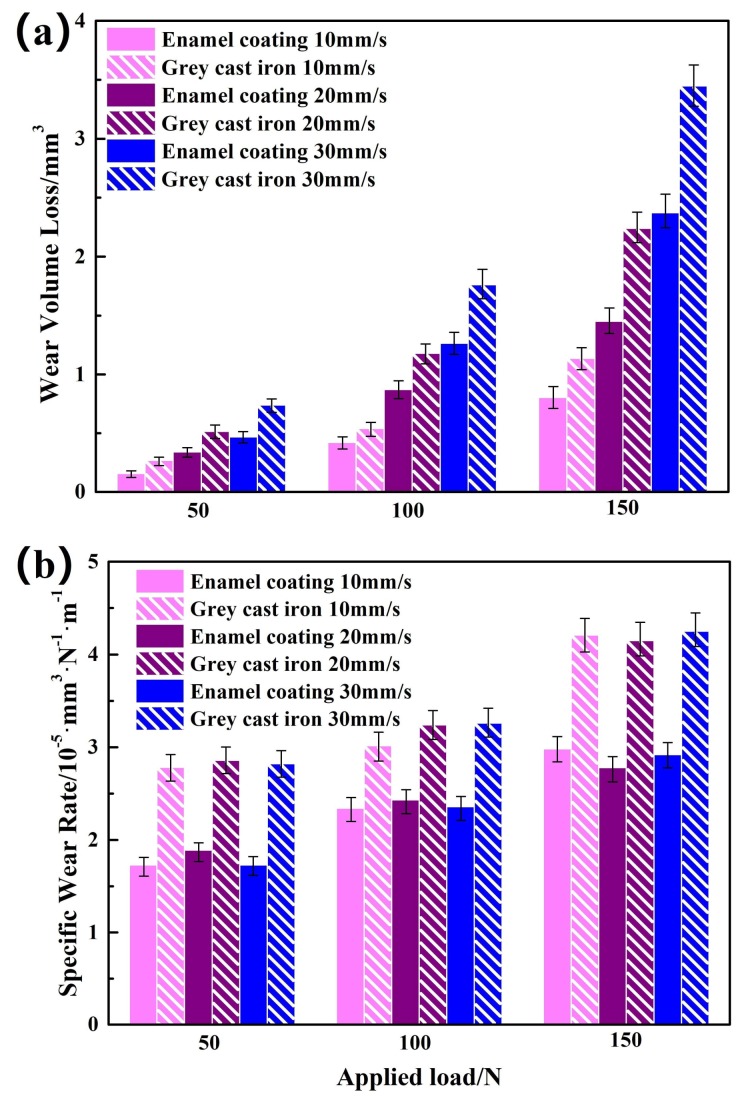
Wear volume loss (**a**) and the specific wear rate (**b**) of the enamel coating and grey cast iron.

Figure 12a shows the wear volume loss of the enamel coating and the grey cast iron at different sliding speed and applied loads. As observed in Figure 11c, the enamel coating had been worn through under the 200 N applied load. Thus, only the wear volume loss of the samples tested at the loads of 50 N, 100 N, and 150 N was calculated and presented in the Figure 12a. Interestingly, the wear volume loss of the enamel coating was always less than that of the grey cast iron, indicating its better wear resistance. Moreover, it could be seen that the wear volume loss of the two kinds of samples increased correspondingly with the increased applied load and sliding speed. Under the same load, faster sliding speed led to the longer sliding distance under the same wear duration, which caused the larger wear volume loss. In the situation of the same sliding speed, the larger loads brought lager friction contact stress, which caused the larger wear volume loss. Similar phenomenon was also reported in the Cr-contained cast iron with increased applied load [28]. Specific wear rate was further used to evaluate the wear resistance of both the enamel coating and the grey cast iron, as shown in Figure 12b. Two conclusions can be extracted from this histogram. On the one hand, the specific wear rate of both samples increased with the increased load. Meanwhile, the specific wear rate of the enamel coating was always lower than that of the grey cast iron, which was about 68%, 78%, and 70% of the grey cast iron at the load of 50 N, 100 N, and 150 N, respectively. On the other hand, there was no obvious difference in the specific wear rate of the both samples at different sliding speed. This phenomenon implied that no obvious change in wear mechanism was resulted by the increased sliding speed, which could be verified by the SEM morphologies of the wear trace (Figure 10 and Figure 11). This weakly speed-depended wear brought into correspondence with the classical Archard’s law and previous reports [29,30]. Based on the above data, one clearly claimed that enamel coating could effectively improve the wear resistance of the grey cast and extend its service life in a complex frictional environment.

## 4. Conclusions

In this study, a low-temperature sintered enamel with high-strength bonding and wear-resistant covering is designed and prepared on a grey cast iron surface. The microstructure characteristics, mechanical properties, and the wear-resisting properties of the optimized enamel coatings were examined, and the following conclusions were drawn:

- The traditional SiO_2_–Al_2_O_3_–B_2_O_3_ composited prescription for an enamel coating was modified to sinter a complete and compact enamel coating at a relatively lower temperature. In the optimized prescription, some quantity of SiO_2_ was used instead of B_2_O_3_, and some of the alkali metals were used instead of Li_2_O. The optimized sintering process of the enamel coating was 730 °C at seven minutes. This coating was composed of both an amorphous phase and a Na_2_Al_2_Si_5_O_14_ crystalline phase.
- The enamel coating sintered by the optimized parameter had a thickness of 150 µm, which uniformly and completely covered the metal substrate with a good gloss. The micro-hardness of the enamel coating was approximately 630 HV_0.2_, which was approximately 2.7 times of the metal substrate. The enamel coating and the grey cast iron substrate had a metallurgical bond, which endowed the sufficient coating bonding stress of approximately 15 MPa.
- The wear mechanism of the enamel coating was a typical brittle exfoliation under the studied friction situation. The wear volume loss and the specific wear rate of the enamel coating were obviously lower than that of the grey cast iron at the different applied load and sliding speed.

## Figures and Tables

**Table 1:** Chemical composition of the raw materials used for enamel.

Component	SiO_2_	B_2_O_3_	Al_2_O_3_	Na_2_O	Li_2_O	ZnO	MgO	Na_2_SiF_6_	CaF_2_	NiO	CoO
**wt/%**	52	14	3.5	6	12	4.4	0.5	3	2	1.6	1

**Table 2:** Chemical composition of the enamel slurry.

Component	Enamel Powder	Ball Clay	Sb_2_O_3_	Borax	Ethyl Alcohol
**Relative Mass Ratio**	100	6	0.2	0.2	70

**Table 3:** Friction test parameters.

Testing Parameters	Value
Friction time/min	30
Sliding stroke /mm	1
Acceleration /mm·s^−1^	0.1
Applied load /N	50, 100, 150, 200
Sliding speed/mm·s^−1^	10, 20, 30
